# Commissioning of a combined stereoscopic x‐ray and surface guided radiotherapy system for performing breath hold gated lung radiotherapy

**DOI:** 10.1002/acm2.70283

**Published:** 2025-11-18

**Authors:** Garrett C. Baltz, Steven M. Kirsner

**Affiliations:** ^1^ Scripps Cancer Center San Diego California USA

**Keywords:** commissioning, deep inspiration breath hold, motion management, stereotactic body radiation therapy, surface guided radiotherapy

## Abstract

**Background:**

ExacTrac Dynamic (ETD) is a novel patient positioning and motion management system for radiation therapy that combines surface guidance, thermal imaging, and stereoscopic x‐rays for image guidance. This combination of technology enables both intrafraction monitoring and surface‐based breath hold gating capabilities.

**Purpose:**

The purpose of this study was to use the American Association of Physicists in Medicine (AAPM) TG‐302 framework to commission two independent ETD systems for performing breath hold gated lung radiotherapy.

**Methods:**

A series of static and dynamic tests were performed using an anthropomorphic thorax phantom placed on a dynamic motion platform. Static tests included localization accuracy and reproducibility, and the impact of reference surface selection on localization. Spatial drift was evaluated during system warmup, during typical treatment session duration, and with changing temperature. Dynamic tests assessed localization accuracy and reproducibility, as well as gating latency. Finally, end‐to‐end testing of a simulated breath hold lung stereotactic body radiation therapy (SBRT) treatment was performed, with both gated and ungated dose measurements being acquired.

**Results:**

Static and dynamic localization accuracy averaged less than 1 mm, with reproducibility within 0.4 mm. An optimal Hounsfield Unit (HU) threshold of‐700 for the reference surface contour was demonstrated to have the best localization accuracy. The maximum observed spatial drift for all tests was less than 1 mm. Congruence between surface guidance, x‐ray imaging, and CBCT were all within 1 mm. Mean beam on and off latency averaged 1023 and 527.1 ms, respectively. End‐to‐end testing demonstrated less than 1% dose difference between gated and non‐gated treatment delivery. The independent ETD systems demonstrated consistent performance across all commissioning tests.

**Conclusions:**

All commissioning tests were within acceptable tolerances published in the AAPM TG‐302 report. This study demonstrated that the ETD system is suitable for performing surface‐guided breath hold gated lung SBRT treatments.

## 1 INTRODUCTION

The use of surface guided radiation therapy (SGRT) for the management of respiratory motion has gained broad adoption in the clinical radiotherapy setting. Survey data from American Association of Physicists in Medicine (AAPM) TG‐324 showed 45% of respondents reported using surface guidance for deep inspiration breath hold (DIBH) in the treatment of left‐sided breast cancer, and 42% reported using SGRT for real‐time monitoring during treatment of thoracic and abdominal sites.^1^ Clinical adoption of SGRT has been enabled by numerous commercially available platforms, with TG‐324 reporting the majority (38%) using AlignRT/OSMS (Vision RT, London, United Kingdom), followed by Catalyst (C‐RAD, Uppsala, Sweden) and Identify (Varian, Palo Alto, CA), with less than 0.5% of respondents reporting using the recently released ExacTrac Dynamic (ETD) system (Brainlab, Munich, Germany).

ETD is a patient positioning and motion management system for linear accelerator‐based radiation therapy. The system consists of a floor mounted stereoscopic x‐ray system and a ceiling‐mounted combination structured light and thermal camera for surface tracking.^2^ This hardware combination makes ETD unique as a unified system capable of performing x‐ray‐based image guided radiation therapy (IGRT) in tandem with SGRT workflows, such as DIBH for breast or thoracic radiation therapy.

In the treatment of thoracic and abdominal sites, while 51% of TG‐324 respondents reported using breath hold for motion management, only 30% of respondents reported using SGRT for respiratory tracking, with the majority still using conventional respiratory signal monitoring. This illustrates a gap in the proportion of clinics using surface guidance for breath hold lung and abdominal treatments as compared to breast, even though SGRT is suitable for breath hold gating of these treatment sites for patients who are candidates for breath hold gated treatment.^3^, ^4^ This discrepancy may be related to lack of literature and experience with using SGRT systems for breath hold gating of these treatment sites.

Critical to the successful clinical implementation of any new technology is performing rigorous commissioning and testing of the system. Due to its unique capabilities, comprehensive commissioning of ETD requires incorporating tests from relevant consensus guidelines applicable to IGRT and SGRT. Guidelines applicable to the x‐ray IGRT functionality include AAPM MPPG 2.b and TG‐142.^5^, ^6^ Recommended tests for SGRT functionality can be found in AAPM TG‐302 and the ESTRO‐ACROP guideline on surface guided radiation therapy.^7^, ^8^ To date, there have been a limited number of studies on the implementation of ETD. Chow et al. measured static positioning accuracy between ETD and cone‐beam computed tomography (CBCT).^9^ Da Silva Mendes et al. characterized the performance of the SGRT system in a static setting.^2^ Perrett et al. outlined a recommended framework for ETD commissioning, including some tests of its dynamic accuracy.^10^ Finally, Goodall et al. performed an image based end‐to‐end testing of using ETD for DIBH breast treatments.^11^ Thus far, there has not been any published literature on commissioning ETD for breath hold gating of thoracic and abdominal treatments. The purpose of this work was to present an SGRT focused commissioning of ETD for the treatment of breath hold gated lung stereotactic body radiation therapy (SBRT), to advance the use of SGRT for these treatments.

## 2 METHODS AND MATERIALS

### 2.1 Commissioning tests and equipment

As the primary aim of this study was to commission ETD for surface‐based breath hold gating for thoracic SBRT treatments, a set of commissioning tests was designed focusing on testing the SGRT functionality of the system for performing this type of treatment. AAPM TG‐302 was the primary guideline used for determining which tests should be included in the commissioning process.^7^ The devised tests could be categorized as static or dynamic tests and are outlined in Table 1.

**TABLE 1:** List of commissioning tests performed.

Static tests	Dynamic tests
Localization accuracy	Localization accuracy
Localization reproducibility	Localization reproducibility
Localization accuracy vs. reference surface selection	Gating latency
Spatial drift during system warmup	End‐to‐end gated dose measurement
Spatial drift during treatment	
Spatial drift vs. Phantom temperature	

**FIGURE 1:**
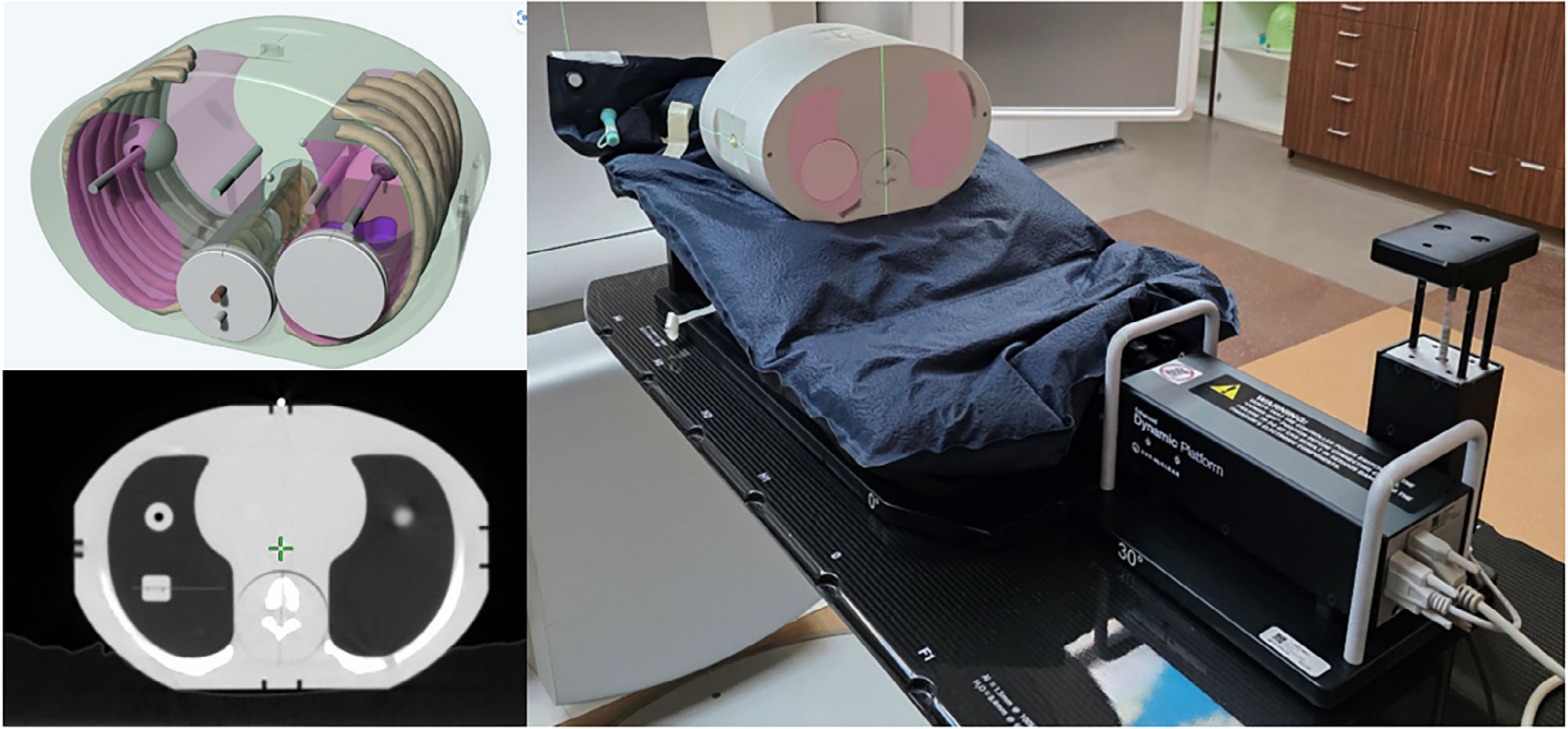
Anthropomorphic thorax phantom and motion platform used for performing commissioning tests.

The phantom used for conducting all commissioning tests was the Sun Nuclear Model 036s E2E SBRT phantom (Sun Nuclear Corporation, Melbourne, FL). This is an anthropomorphic thorax phantom containing ribs, spinal column, heart, and lungs with embedded tumor volumes with cutouts for an ionization chamber. All materials are tissue equivalent, making the phantom radiographically visible as well as being suitable for performing dosimetric measurements. To enable performing dynamic motion tests with the phantom, the phantom was placed on a Sun Nuclear Enhanced Dynamic Platform Model 008PLX. This is a software controlled programmable motion platform with sub‐millimeter accuracy. The software allows for custom respiratory cycles and waveforms to be programmed to control the platform movement. The motion platform was configured with the incline plane orientation, and the thorax phantom was immobilized on the platform using a vacuum cushion (Innovative Oncology Solutions, Memphis, TN), shown in Figure 1. Positioning the phantom on the inclined plane enabled the movement of the phantom in both the inferior‐superior and posterior‐anterior directions, allowing for the simulation of a breath hold motion.

Commissioning tests were performed on two independent ETD systems. This allowed for double data collection for each test, as well as the opportunity to assess intersystem variability. ETD System 1 was installed on a Varian TrueBeam Edge (Varian Medical Systems, Palo Alto, CA), and ETD System 2 was installed on a Varian TrueBeam STx. The ETD systems were running software version 1.1.

### 2.2 Static tests

A CT scan of the stationary phantom setup shown in Figure 1 was acquired using a GE Optima 580 CT scanner (GE Healthcare Medical Solutions, Chicago, IL). The institution standard lung SBRT protocol was used: helical mode, 120 kVp, 500 mA, 1.25 mm slice thickness, and 60 cm field‐of‐view. A mock radiation treatment plan was made using the CT scan in the Varian Eclipse v15.6 treatment planning system (TPS). The phantom was scanned with a 3‐point setup marked with radiopaque ball bearings (BBs) that was set as the marked isocenter in the TPS. A final treatment isocenter was placed in the small target volume in the right lung of the phantom. The RT plan, CT images, and the RT structure set were exported to ETD and prepared in the software to be used for conducting the static tests.

#### 2.2.1 Static localization accuracy versus reference surface selection

As discussed in section 3.4.1 of AAPM TG‐302, the reference surface selection can affect the localization accuracy of SGRT systems.^7^ ETD relies on an external body contour created in the TPS as the reference surface for patient prepositioning, as well as the reference surface for breath hold treatments. External contours are typically created automatically using a Hounsfield Unit (HU) threshold operation. To assess how different HU thresholds used to create the reference surface affect the localization accuracy, eight different automatically segmented external body contours were created for the phantom plan by varying the HU threshold from −200 to −900 HU in 100 HU increments.

On the treatment machine, the thorax phantom was first aligned using the lasers to the marked isocenter, which was used as a consistent starting location between trials and between the two different systems. ETD was used to perform prepositioning, which uses the surface camera and reference surface to assess the current phantom position and generate shifts to position the phantom at final treatment isocenter. After the phantom was shifted using prepositioning, x‐ray images were acquired using ETD, and the residual positioning error to the final isocenter was recorded. With the phantom in the same position, a CBCT was acquired, and the residual positioning error was recorded. This was repeated a total of three times for each of the eight different HU threshold reference surfaces. The lowest average residual positioning error and best agreement between x‐ray and CBCT positioning were used to determine the optimal HU threshold to use for the external body contour reference surface.

#### 2.2.2 Static localization accuracy

The purpose of this test was to assess the localization accuracy of the SGRT system. Based on the results of the reference surface analysis, an HU threshold of −700 was determined to be the best value for creating the external body reference surface and was the reference surface used for all other tests in the current study. Localization accuracy was assessed by using prepositioning to set up the thorax phantom to the marked isocenter and then applying a range of manual offsets. Shifts of up to ± 10 cm in the longitudinal and lateral directions, and up to ± 5 cm in the vertical direction were applied, as well as up to a 3‐degree yaw couch rotation. After the known offset was applied, ETD x‐rays and a CBCT were acquired. Deviations from the known applied offset were recorded for each of the surface/thermal monitoring, x‐ray imaging, and CBCT.

#### 2.2.3 Static localization reproducibility

Localization reproducibility was assessed by using the SGRT system to perform multiple trials of the same phantom positioning. The thorax phantom was aligned to marked isocenter, and couch coordinates at this location were recorded. SGRT prepositioning was used to generate shifts to the final isocenter, which were recorded and applied. At the final isocenter position, ETD X‐ray images and a CBCT were acquired, and residual positioning errors were recorded. The phantom was then moved back to the initial couch positioning at marked isocenter, and the test was repeated 10 times.

#### 2.2.4 Spatial drift during system warmup

It is well documented that SGRT systems can exhibit spatial drift when the system is first turned on and require a warmup period before the system stabilizes enough for clinical use. In order to assess the spatial drift during warmup of ETD, the system was powered down overnight. The system was then turned on the next morning, and the mandatory ETD daily quality assurance (QA) was quickly performed. The thoracic phantom was immediately set up and positioned at the treatment isocenter. A tracking region of interest (ROI) covering the entire phantom surface area was defined, and surface/thermal monitoring was enabled. The drift was monitored and recorded for a total time of 40 min to determine when the drift stabilized.

#### 2.2.5 Spatial drift during treatment

Even with an SGRT system that has been properly warmed up, spatial drift can still occur as the cameras heat up during active surface monitoring. The purpose of this test was to assess the spatial drift that can occur during an average patient treatment session. The methodology for this test was identical to the system warmup test presented in Section 2.2.4. Before commencement of the test, the system had been powered on for multiple hours and was fully warmed up. Surface monitoring was recorded for 30 min, representing a typical treatment session.

#### 2.2.6 Spatial drift versus phantom temperature

A unique feature of the ETD system is the incorporation of a thermal camera for SGRT monitoring. The thermal camera records the patient's unique heat signature and correlates it with the reconstructed 3D surface structure to create a “4D” hybrid thermal surface to monitor. The use of thermal information in ETD surface monitoring requires testing of the spatial drift with temperature change. This test was specifically designed to assess the impact of temperature change on spatial drift. The methodology previously presented in Sections 2.2.4 was used for this test. Prior to performing the test, the phantom was placed in a blanket warmer set at 38°C for 1 h. Upon completion of warming, the phantom was set up and the surface monitoring was initiated. The change in temperature of the phantom was periodically recorded with a thermometer in contact with the phantom as the phantom naturally cooled in the treatment room environment with an ambient temperature of ∼22°C. Monitoring was performed for 40 min, and the spatial drifts were recorded.

### 2.3 Dynamic tests

**FIGURE 2:**
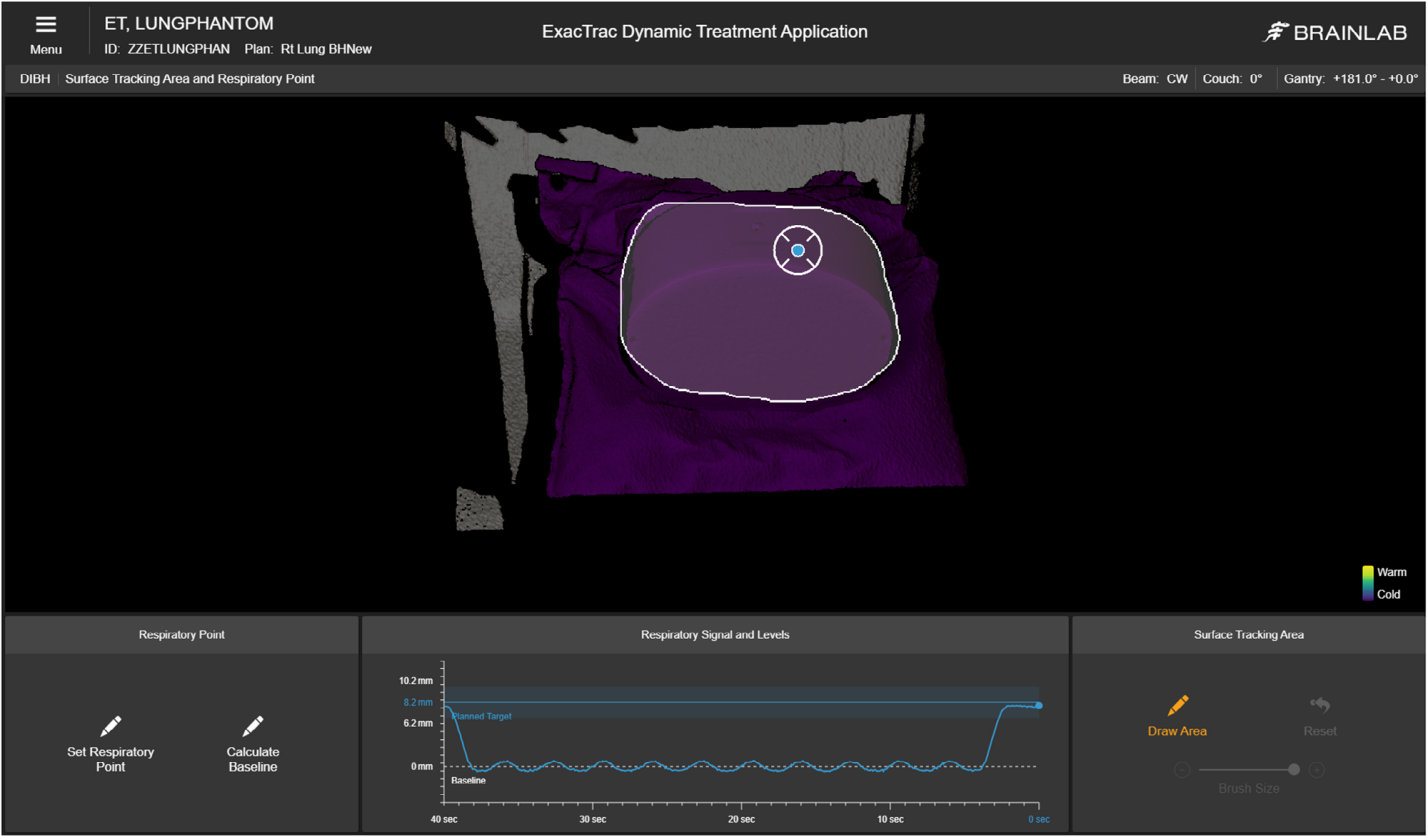
Surface tracking area and location of the respiratory point location (blue dot) on the phantom used for gating.

Testing of the dynamic localization capabilities of ETD was performed using the thorax phantom placed on the dynamic motion platform to simulate a breath hold treatment. The motion platform control software was used to create a mock breath hold breathing pattern consisting of 10 cycles of shallow respiration over 15 s followed by a simulated deep inspiration breath hold for 12 s, presented graphically in Figure 2. This motion program resulted in a 0.8 cm movement of the phantom in the anterior‐posterior axis, with a 4 cm movement in the superior‐inferior axis. CT scans of the phantom were acquired using the previously described lung SBRT protocol during both the free‐breathing and breath hold cycles of the motion program. These scans were imported into the TPS for the creation of a lung SBRT treatment plan. The small tumor volume containing the ionization chamber cutout in the right lung of the phantom was used as the target. External body contours were created using an HU threshold of ‐700 HU for both the free‐breathing and breath hold scans. The free‐breathing body contour was copied to the registered breath hold CT dataset and named with the proper nomenclature to be used for the initial pre‐positioning and breath hold guidance on ETD. The institution standard lung SBRT protocol was used for treatment planning on the breath hold CT: 1250 cGy/fraction delivered using VMAT with two ipsilateral 180° arcs and a beam energy of 6X‐FFF with a maximum dose rate of 1400 MU/min. Dose was calculated on a 1.25 mm dose grid using the AcurosXB algorithm, reporting dose‐to‐medium. The final treatment plan was exported to ETD and configured to use ETD‐based breath hold gating.

#### 2.3.1 Dynamic localization accuracy and reproducibility

ETD uses the free‐breathing and breath hold reference body contours to automatically set the target threshold for patient feedback to achieve the same breath hold level established during CT simulation. High dynamic accuracy and reproducibility of the ETD SGRT system are critical to achieve reproducible breath holds and accurate target localization. Dynamic localization accuracy was tested by comparing the ETD measured breath hold level of the phantom to the known level measured from the free‐breathing and breath hold CTs. The thorax phantom placed on the motion platform was positioned on the treatment couch, and the programmed breathing motion was initiated. Prepositioning was used to shift the phantom to the treatment isocenter. Once the ETD system established the baseline respiration level, the respiratory point used for threshold targeting and gating was set at stable location, shown in Figure 2. Treatment monitoring was then initiated, and when the motion program entered the breath hold stage, the measured breath hold level was recorded. In addition, while the phantom was in the breath hold position, the residual errors of the surface tracking area were recorded. This process was repeated a total of 10 times, with the results recorded for each trial.

#### 2.3.2 Gating latency

Low latency in interrupting the treatment delivery when the patient moves out of the gating window is a critical performance requirement for gating systems to maintain dosimetric accuracy in gated treatments. This test was designed to measure the latency of the ETD system to terminate the treatment delivery when the patient falls out of breath hold. For this test, the phantom setup and gating methodology described in Section 2.3.1 were used. The smallest allowable gating window of ± 0.5 mm of the respiratory target threshold was set so ETD would gate the treatment as quickly as possible when the phantom moved out of breath hold. To measure the latency, while the motion program was running, a high‐speed camera simultaneously recorded the monitors of the motion control software, the ETD console, and the linac treatment console. The camera used was a Samsung Galaxy S24 Ultra cellphone (Samsung Electronics Co., Suwon, South Korea) recording with a video resolution of 1920 × 1080 at 120 frames per second, allowing for a time resolution of 8.3 ms. A script was developed in Python v3.8 to determine the number of frames between three key events in the video feed: the motion control signal initiating phantom movement out of breath hold, the ETD system triggering an out‐of‐breath‐hold state, and the linac console's beam‐on indicator turning off. Calculating the time between these three events allowed for a measurement of the total system beam off latency from when the phantom exited breath hold to treatment interruption, as well as the latency of communication between ETD and the linac to gate treatment delivery. Using the same methodology, the total beam on latency was also calculated based on the difference from the phantom first entering the gating window to when the beam on indicator was initiated on the linac, as well as the latency of the beam on signal being sent from ETD to the linac. Ten separate breath hold cycles were recorded, and the latency times were calculated for each trial.

#### 2.3.3 End‐to‐End gated dose measurement

To assess the dose delivery accuracy using ETD for breath hold gating, an end‐to‐end test of a breath hold gated lung SBRT treatment was performed. The thorax phantom and motion platform were positioned on the treatment table, and prepositioning was used to align the phantom to treatment isocenter, after which the motion program was initiated. While in breath hold, x‐rays were acquired with ETD, and the residual shifts were applied to position the phantom at treatment isocenter. The respiratory point location was defined as in Figure 2, and a ± 2 mm gating window was set on the breath hold target threshold. An ADCL calibrated Sun Nuclear SNC125c small volume ionization chamber was placed in the cutout in the center of the target volume and connected to a calibrated Sun Nuclear PC Electrometer for charge collection. The measured charge was converted to dose using Equation 1, using standard AAPM TG‐51 nomenclature.^12^ The beam quality conversion factor $\left(k_{Q}\right)$ for the ionization chamber was calculated using Table 6 in Alissa et al.^13^

(1) $$Dose\;\left(\mathrm{cGy}\right)\;=\;M\;\times N_{D,w}\times \;\;k_{Q}\times P_{elec}\times P_{TP}$$

Three separate dose measurements were performed. The first measurement was with the phantom statically in the breath hold position, representing a baseline ungated dose delivery. Two other dose measurements were performed with the phantom moving in the previously described programmed breathing motion, where ETD would gate the beam when the phantom was not in breath hold. One measurement was performed using a total of 8 breath hold cycles for complete treatment delivery, and the other using a total of 14 breath hold cycles. The gated dose measurements were compared to the ungated delivered dose measurements, and to the TPS calculated mean dose to the chamber volume.

## 3 RESULTS

### 3.1 Static localization accuracy versus reference surface selection

**FIGURE 3:**
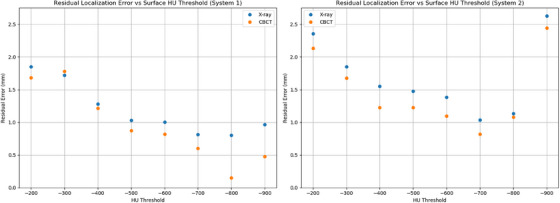
Residual localization error versus reference surface HU threshold for both systems. HU, Hounsfield Unit.

Figure 3 shows the results for residual localization error (overall displacement) for both CBCT and ETD x‐ray images versus the HU threshold used to create the reference body contour. The lowest residual error and best agreement between x‐rays and CBCT were observed for an HU threshold of −700. This threshold resulted in residual errors of less than or equal to 1.0 mm and agreement of CBCT and x‐ray localization within 0.2 mm. These results were consistent for both systems that were tested.

### 3.2 Static localization accuracy

**FIGURE 4:**
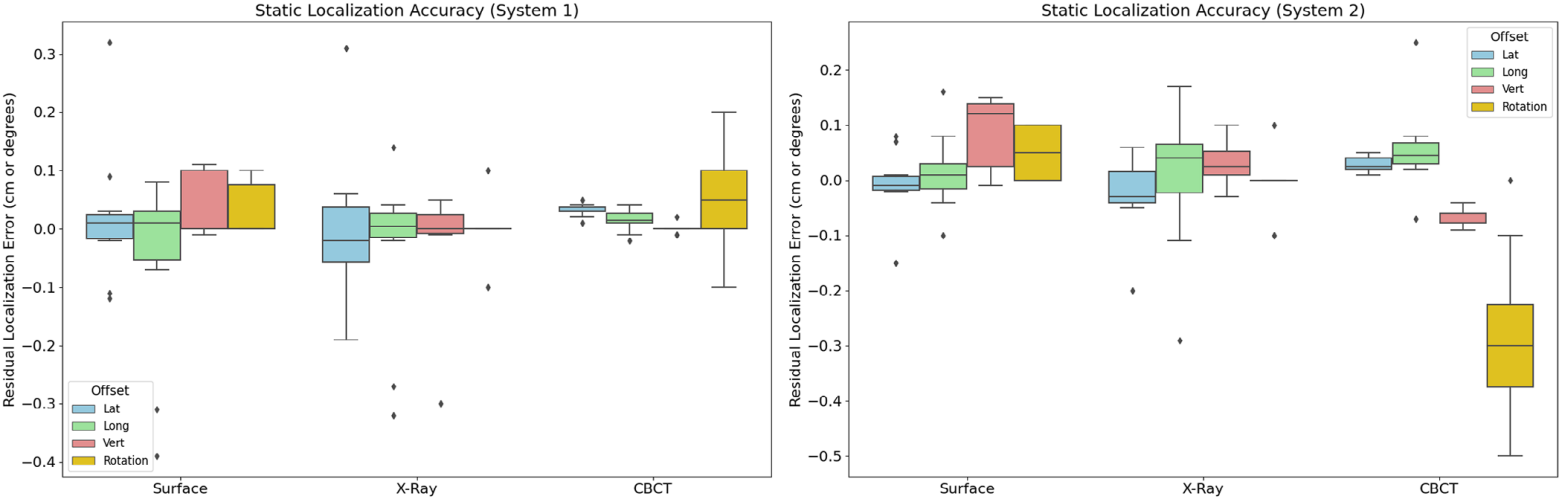
Static localization accuracy calculated as residual error from known applied shift.

Box and Whisker plots of the residual localization error for the static localization accuracy tests are presented in Figure 4. Overall displacements applied to the phantom ranged from 28.3 to 113.1 mm. The mean localization error for the surface/thermal system was less than 1.0 mm for all translational directions and less than 0.1 degrees yaw rotation for both systems tested. This was comparable to the localization accuracy of the x‐ray and CBCT systems, with mean errors of 0.9 and 0.7 mm, respectively. Maximum errors of 3.2 mm for surface and x‐ray localization were observed. However, this was only in situations when the overall displacement of the phantom exceeded 100 mm and a 1 degree or greater yaw rotation was applied. With no rotation applied, surface maximum localization error was within 1.0 mm.

### 3.3 Static localization reproducibility

**FIGURE 5:**
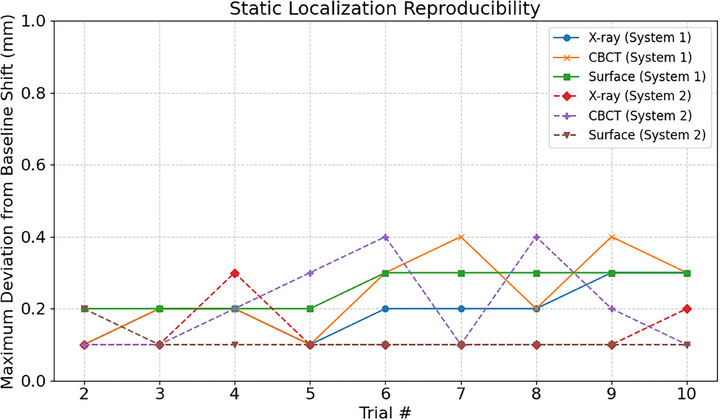
Static localization reproducibility for both systems showing maximum deviation from the initial baseline shift.

Static localization reproducibility was quantified using the maximum translational deviation in residual positioning error from the initial localization trial. The results of this testing are presented in Figure 5. For System 1, surface‐based localization reproducibility was within 0.3 mm for all translational movements and within 0.1 degrees for all rotational axes. Maximum deviation in positioning for the x‐ray and CBCT imaging was 0.3 and 0.4 mm, respectively, with maximum rotational deviations of less 0.1 degrees. System 2 demonstrated reproducibility consistent with System 1, as shown in Figure 5.

### 3.4 Spatial drift during system warmup

**FIGURE 6:**
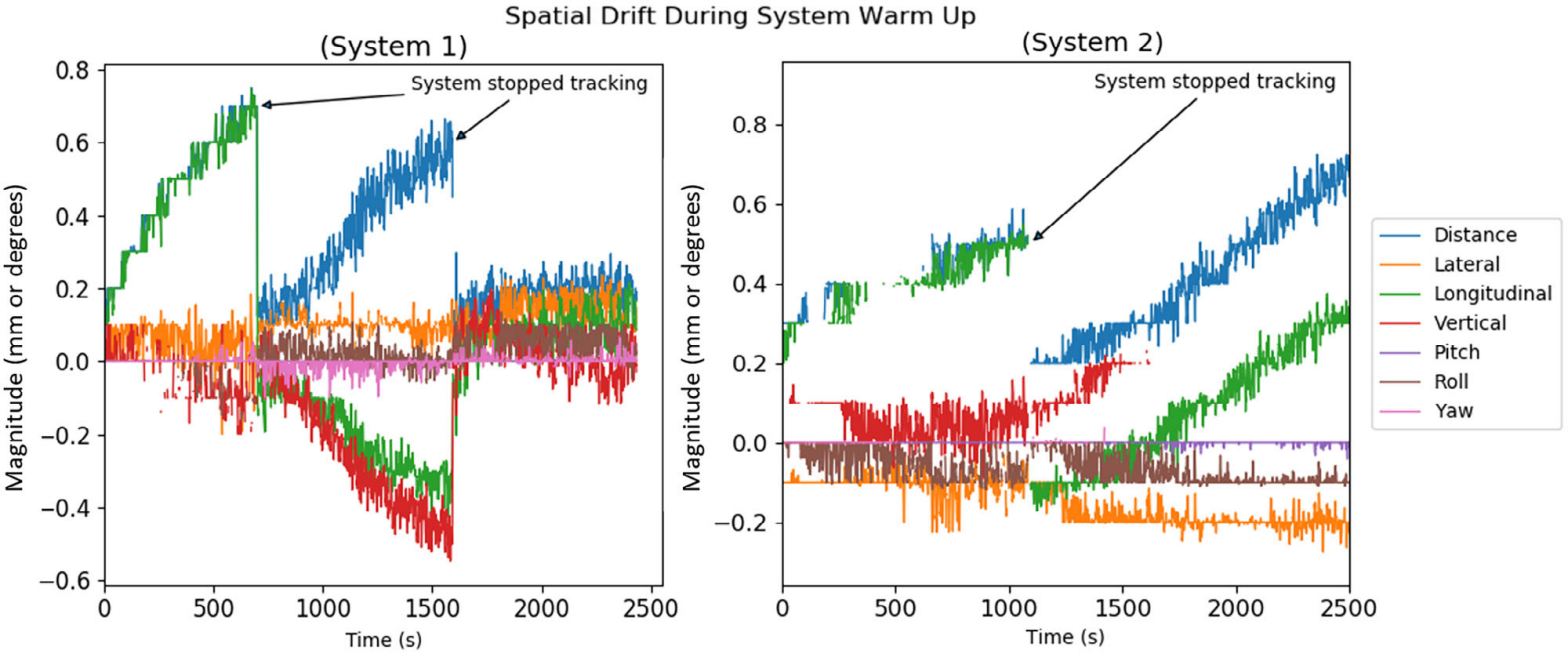
Plots of spatial drift of surface monitoring during system warmup.

Plots of the spatial drift recorded during system warmup for both systems are presented in Figure 6. As the systems were warming up, both systems automatically stopped tracking after 11 and 27 min for System 1, and after 17 min for System 2. This is an automated feature of the ETD system that detects when the surface tracking has become unreliable and will prompt the user to reacquire a surface baseline. For both systems, total recorded drift was less than 0.8 mm even before the systems stopped tracking. Spatial drift for System 1 was stable after tracking was restarted at 27 min, and System 2 maintained tracking with overall spatial drift less than 0.7 mm up to the conclusion of the test. The maximum rotational drift observed for both systems was 0.2 degrees. The manufacturer recommends a warmup time of 1 h, and the system has an automated start feature that can be configured to allow the system to be fully warmed up before clinical treatments begin for the day. The results of this test indicate that the recommended warmup time is sufficient for the SGRT system to stabilize before clinical use.

### 3.5 Spatial drift during treatment

**FIGURE 7:**
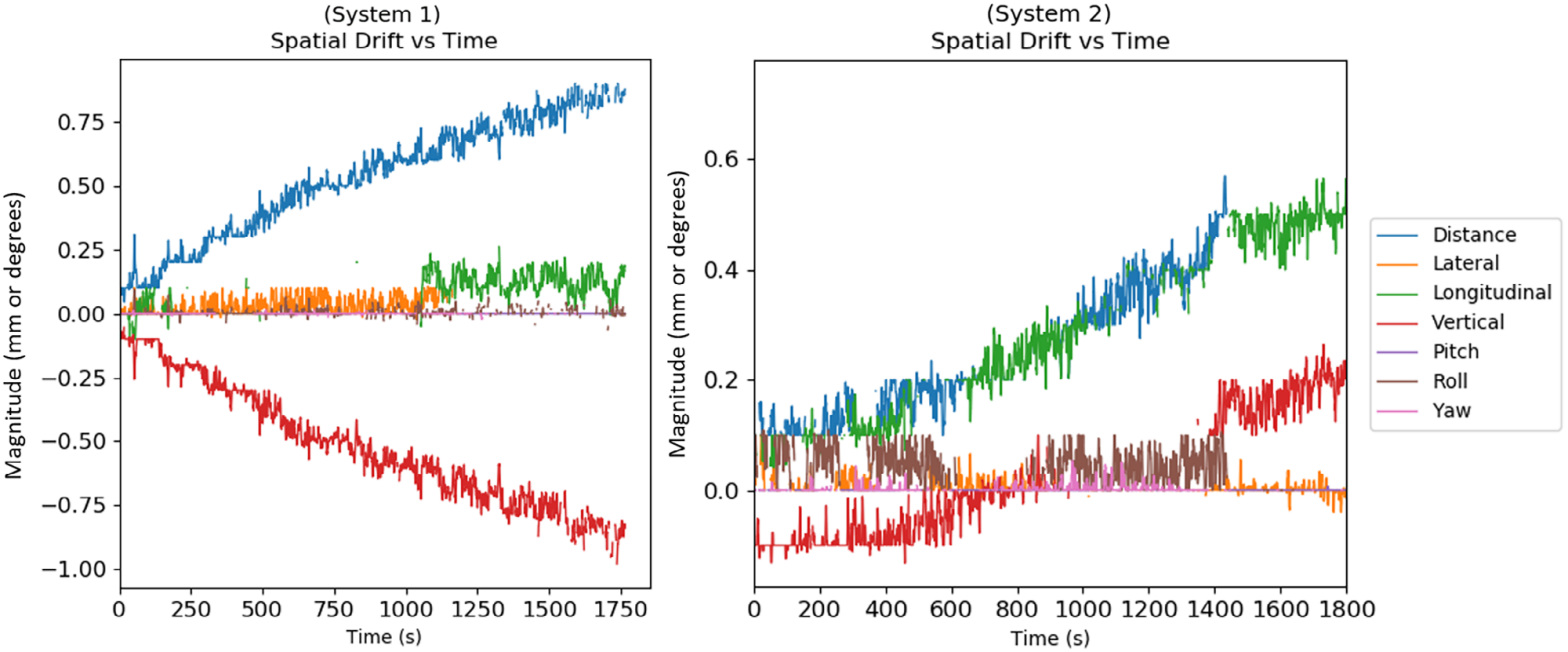
Plots of spatial drift versus time for a typical treatment session with the system fully warmed up.

Plots of the spatial drift recorded during a typical treatment session with the system fully warmed up are presented in Figure 7 for both systems. Maximum translational drift was 0.85 mm for System 1 and 0.5 mm for System 2. The direction of drift was primarily in the vertical direction for System 1, while primarily in the longitudinal direction for System 2. Maximum rotational drift was less than 0.1 degrees for both systems. Neither system exhibited instability nor stopped tracking during monitoring.

### 3.6 Spatial drift versus phantom temperature

**FIGURE 8:**
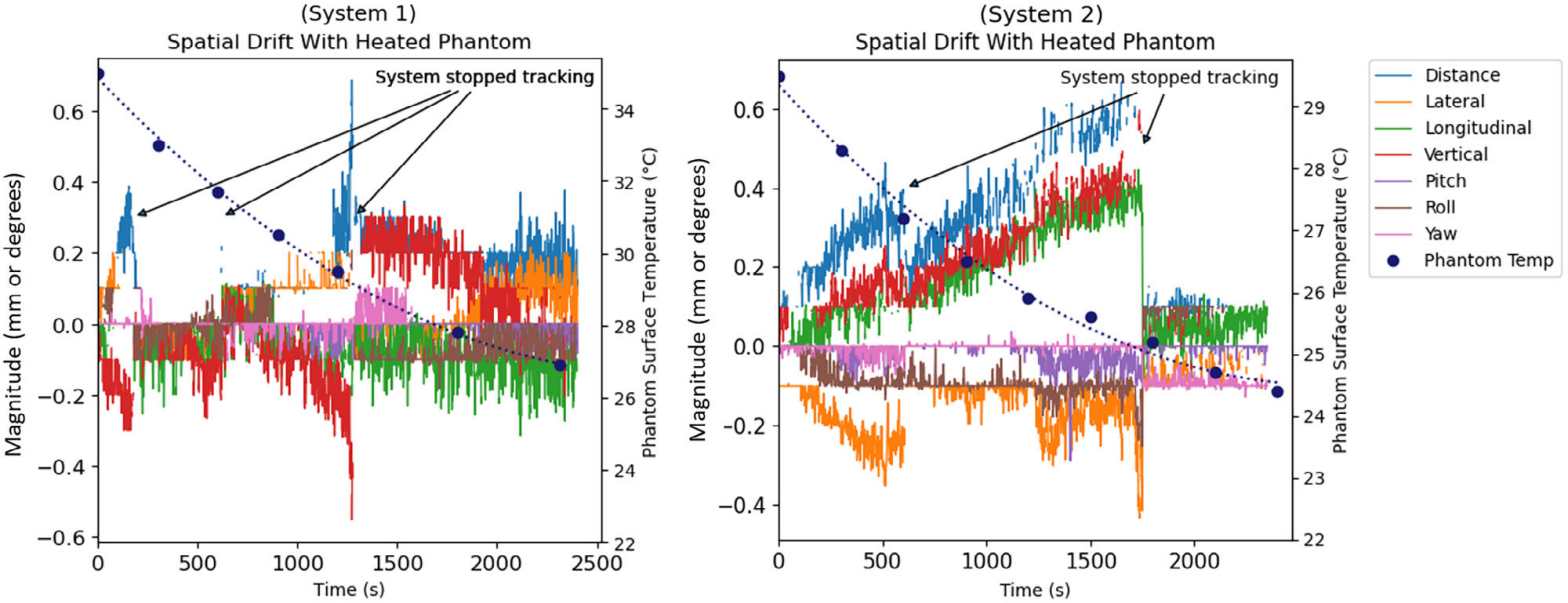
Plots of spatial drift and phantom surface temperature versus time.

Plots of the spatial drift and phantom surface temperature versus time are illustrated in Figure 8. Both systems exhibited a maximum translational drift of 0.6 mm and a rotational drift of less than 0.1 degrees. As observed in the system warmup spatial drift testing in Section 3.4, both systems had times where monitoring was automatically stopped due to the tracking becoming unreliable. This occurred three times for System 1 and twice for System 2. The systems' stopping tracking appeared to correlate to when the change in temperature of the phantom exceeded approximately 3°C. This is most likely due to the thermal heat signature of the phantom changing too much compared to when the reference was acquired at the start of monitoring.

### 3.7 Dynamic localization accuracy and reproducibility

The ETD measured breath hold level was within 0.3 mm of the expected level of 8 mm for both systems, measuring 7.6 mm for System 1 and 7.7 mm for System 2. Measurement of the breath hold level was also highly reproducible, with the measured level identical for all 10 trials on both systems. Localization accuracy and reproducibility of the breath hold surface tracking area compared to the reference breath hold were also good, with a mean (± SD) residual translational error of 0.3 ± 0.2 mm and 0.07 ± 0.04 mm for Systems 1 and 2, respectively. Maximum rotational residual error was 0.1 degrees for both systems.

### 3.8 Gating latency

Average (± SD) total beam off system latency between the motion signal being sent and treatment interruption was 525 ± 188.6 ms for System 1 and 529.2 ± 98.5 ms for System 2. This measurement is inclusive of any latency between the motion control software signal and the motion platform starting motion. Latency between the ETD system and the linac for the beam off signal averaged 66.7 ± 40.8 ms for System 1 and 69.4 ± 31.1 ms for System 2. Total beam on latency averaged 1009.7 ± 64.8 ms for System 1 and 1036.1 ± 55.6 ms for System 2. Latency between the ETD system and the linac for the beam on signal averaged 87.5 ± 47.6 ms for System 1 and 119.4 ± 37.8 ms for System 2.

### 3.9 End‐to‐End gated dose measurement

Results of the end‐to‐end mock lung SBRT treatment dose measurements acquired on both systems are presented in Table 2. Measured dose was within 3.5% of the TPS calculated dose for all measurement trials, for both gated and ungated beam delivery. In examining the effect of using ETD for breath hold gating on treatment delivery, gated dose measurements agreed very well with the respective ungated dose measurements, with both systems’ gated dose within 0.9% of the measured ungated dose. Furthermore, the number of breath holds required to complete the full treatment delivery also did not demonstrate an effect on the treatment delivery, with the measured dose for the 14 and 8 breath trials within 0.8% and 0.5% for System 1 and 2, respectively.

**TABLE 2:** Dose measurements comparing ungated to ETD breath hold (BH) gated treatment delivery for the end‐to‐end phantom test.

	Trial	Dose (cGy)	Ratio Measured/Expected	Ratio Gated/ Ungated
	TPS expected dose	1222.3	‐	‐
System 1	Ungated	1264.1	1.034	‐
	ETD BH gated (14 breaths)	1262.4	1.033	0.999
	ETD BH gated (8 breaths)	1253.3	1.025	0.991
System 2	Ungated	1258.8	1.030	‐
	ETD BH gated (14 breaths)	1264.5	1.035	1.005
	ETD BH gated (8 breaths)	1259.1	1.030	1.000

Abbreviation: ETD, ExacTrac Dynamic; TPS, treatment planning system

## 4 DISCUSSION

The results of the commissioning tests performed in this study indicate that the overall performance of the ETD system was found to be within tolerances outlined in AAPM TG‐302. Static localization accuracy was within 1.0 mm when only translational shifts were applied, and applied rotational shifts agreed within 0.1 degrees. Compared to previous literature on ETD, Perrett et al. observed maximum translational deviations of 0.3 mm,^10^ Chow et al. observed an average error of 0.57 mm^9^ and Goodall et al. reported average error of 1.0 mm.^11^ Despite TG‐302 recommending localization accuracy be examined over a ± 100 mm offset from isocenter, existing ETD literature has only reported investigating accuracy for offsets in the range of ± 20 mm.^9^ Da Silva Mendes et al. investigated offsets of up to 65.6 mm and reported a maximum deviation of 1.5 mm for a cold phantom and 1.3 mm for a warm phantom.^2^ For the current study, maximum positioning errors of up to 3.2 mm were observed when the displacement of the phantom exceeded 100 mm and a rotational yaw shift of 1 degree or greater was applied. Although this technically exceeds recommended tolerances, positioning accuracy for this large an offset would only be clinically relevant for surface‐guidance based initial positioning of the patient, which would typically be followed by IGRT‐based positioning.

Only one previous study has investigated the effect of reference surface generation on the static localization accuracy of ETD prepositioning. Goodall et al. investigated the positioning error for a breast, pelvis, and head phantom using reference surfaces created with HU thresholds ranging from −600 to 200.^11^ The authors reported that no optimal HU value could be determined for all phantoms, and that HU values between −600 and −200 provided adequate positioning. However, it did appear that an HU threshold of −600 had the lowest longitudinal positioning error for all the phantoms and comparable errors in the lateral and vertical directions based on Figure 3 in their work. These results are in agreement with the current study, which found a threshold of −700 to provide the lowest positioning error and best agreement with x‐ray and CBCT positioning.

The spatial drift of the system was found to be less than 1 mm, both during system warmup and during a typical treatment session duration, which is within recommended tolerances. This is also in agreement with previous literature, with Perrett et al. reporting that drift was ≤ 1 mm during both warmup and a 5 min duration.^10^ Da Silva Mendes et al. investigated spatial drift over a 70 min duration and reported a maximum drift of 0.4 mm for a warm phantom and 0.8 mm for a cold phantom.^2^ The cold phantom results match closely to the current study, with a maximum observed spatial drift of 0.85 mm. While previous works investigated spatial drift for both constant temperature warm and cold phantoms, the current study is the first to investigate drift versus change in temperature of the phantom over time. As the phantom cooled, overall spatial drift was observed to be ≤ 0.6 mm, however the system would stop tracking once the change in temperature exceeded 3°C. This is unlikely to occur clinically, as a patient's body temperature would not change with this magnitude over a normal treatment session. However, it has been observed that warmed blankets placed on a patient can cause the surface tracking to stop as the blanket cools if the blanket is included in the tracking area. For this reason, it is institutional policy to exclude blankets from the surface tracking area, which has prevented surface tracking being lost during treatment.

Commissioning of ETD for breath hold lung treatments was the primary aim of the study, and the dynamic tests were an integral component of assessing the performance of the system for use in surface‐based gating. ETD demonstrated high dynamic localization accuracy, with the measured breath hold level within 0.3 mm of ground truth. Similarly high localization accuracy and reproducibility of the breath hold surface tracking area were observed, with a mean residual translational error of 0.3 ± 0.2 mm. The only previous literature reporting on ETD dynamic localization accuracy was Goodall et al., who measured the deviation of a BB embedded in a breast phantom going into breath hold. They reported average deviation of the BB from planned location over five trials of 1.0, 0.4, and 0.1 mm in the lateral, longitudinal, and vertical directions, respectively.^11^ Critical to the clinical efficacy of any gating system used in radiotherapy is verification that the gating of the treatment delivery does not alter the dose delivered compared to an ungated delivery. Low gating latency is required to maintain dosimetric accuracy, and this is the first study to measure latency of the ETD system coupled with a Varian linac. Mean latency between ETD and the linac was measured to be 67–69 ms for beam off and 88–119 ms for beam on, which is within the ESTRO‐ACROP recommended lag time tolerance of 200 ms.^8^ AAPM TG‐302 recommended tolerances were also met, with a total latency time of < 1 s for DIBH workflows.^7^ This study demonstrated a significantly longer total beam on latency of 1010–1036 ms compared to the total beam off latency of 525–529 ms. This was expected as the ETD system is configured to add a 1000 ms delay to initiating beam on from when the gating threshold is reached to allow for the patient anatomy to settle in the breath hold. Gating latency for ETD determined in the current study is comparable to previously reported latencies for SGRT systems coupled to Varian linacs. Barfield et al used a cine imaging technique to measure a beam on latency of 320–900 ms and a beam off latency of 290–940 ms for AlignRT, depending on the frame rate mode used.^14^ Chen et al reported beam on and off latencies of 303 and 34 ms, respectively, for the Catalyst system based on translational film profile irradiations.^15^ Clinically acceptable gating performance was further supported by the end‐to‐end dose measurements, which to the authors’ knowledge are the first reported ETD breath hold gated dose delivery results. Dose measurements confirmed that gated dose delivery was within 0.9% of the ungated dose delivery. These results demonstrate the ETD system performs within the AAPM TG‐302 tolerances of ≤ 2% dose change for beam hold.

There are several limitations of the current study related to recommended tests for SGRT systems that were not performed. One test not performed was the effect of skin tone on any of the tests conducted. All tests were only performed with the E2E thorax phantom, which could be considered closest to a light skin tone. Perrett et al. previously investigated the effect of skin tone on ETD surface tracking and found that it did not alter the accuracy of the system.^10^ The effect of the surface tracking ROI size on spatial accuracy or system latency was also not investigated. While conventionally there may be a tradeoff of accuracy and latency based on the size of the tracking ROI for most SGRT systems, this test has limited relevance due to how the ETD system operates, as the minimum required ROI size is directly influenced by the topology of the surface being tracked. A flatter surface, such as typically observed in the thorax region, offers less surface information, prompting the system to require a relatively larger ROI to ensure reliable tracking. Based on institutional clinical experience, the ROI size used for the phantom in the current study is representative of what is required clinically for treatments in the thorax, making the presented spatial accuracy and latency results relevant for that application. Because significantly different ROI sizes would not be able to be used clinically for this treatment site, the effect of different ROI sizes was not investigated.

The performance characteristics of ETD reported in this study could be considered when determining PTV margins to use for treatment. Localization accuracy and reproducibility of the SGRT system will factor in as fundamental limits on overall achievable target localization accuracy. However, patient related uncertainties such as inter‐breath hold variation and intrafraction motion are likely to be more significant and ultimately drive what PTV margin is appropriate.

Unique to this study was performance of a uniform set of commissioning tests on two independent ETD systems. In addition to doubling the amount of data collected for analysis, this also allowed for a comparison of the inter‐system performance of ETD. The results demonstrated that the two systems had comparable performance within recommended AAPM TG 302 tolerances. This provides additional confidence in the applicability of the findings of this study, such as the ‐700 HU threshold, to not only additional ETD systems that get deployed within the institution, but also to the broader clinical community of ETD users.

## 5 CONCLUSION

This study commissioned two independent ETD systems for breath hold lung treatments using an AAPM TG‐302 framework. A series of static and dynamic commissioning tests were performed. Static tests included localization accuracy and reproducibility, the impact of reference surface selection on localization, and spatial drift during warmup, treatment, and varying temperature. Dynamic tests assessed localization accuracy and reproducibility, and included an end‐to‐end phantom test of a breath hold lung SBRT treatment. Both commissioned ETD systems met all tolerances specified in the AAPM TG‐302 report. Static and dynamic localization accuracy were within 1 mm, and spatial drift was < 1 mm in all testing conditions. An optimal HU threshold of ‐700 was determined for creating reference surface contours, yielding the lowest residual localization errors and the best agreement with external imaging. End‐to‐end testing of a phantom breath hold gated lung SBRT demonstrated agreement between delivered and planned doses within 3.5%, and the gated dose to be within 0.9% of the ungated dose. These commissioning results indicate that ETD can be safely used for breath hold gated radiotherapy of thoracic treatment sites.

## AUTHOR CONTRIBUTION

Garrett C. Baltz and Steven M. Kirsner are equally responsible for the study design, acquisition and analysis of the data, and writing of the manuscript.

## CONFLICT OF INTEREST STATEMENT

Scripps Cancer Center is a reference site for Brainlab products. Scripps Cancer Center is a Sun Nuclear Patient Safety Center of Excellence and is a reference site for Sun Nuclear products.
